# Deletion in the Promoter of *PcPIN-L* Affects the Polar Auxin Transport in Dwarf Pear (*Pyrus communis* L.)

**DOI:** 10.1038/s41598-019-55195-7

**Published:** 2019-12-09

**Authors:** Xiaodong Zheng, Haiyue Zhang, Yuxiong Xiao, Caihong Wang, Yike Tian

**Affiliations:** 1 College of Horticulture, Qingdao Agricultural University, Qingdao, 266109 China; 2 Qingdao Key Laboratory of Genetic Improvement and Breeding in Horticulture Plants, Qingdao, 266109 China

**Keywords:** Plant development, Plant molecular biology

## Abstract

Dwarf cultivars or dwarfing rootstocks enable high-density planting and are therefore highly desirable in modern pear production. Previously, we found that the dwarf growth habit of pear is controlled by a single dominant gene *PcDw*. In this study, *PcPIN-L* (*PCP021016*) was cloned from dwarf-type and standard-type pears. *PcPIN-L* expression was significantly lower in the dwarf-type pears than in standard-type pears, which was caused by the CT repeat deletion in the promoter of dwarf-type pears. *PcPIN-L* overexpression in tobacco plants enhanced the growth of the stems and the roots. Notably, the indole acetic acid (IAA) content decreased in the shoot tips and increased in the stems of transgenic lines compared with wild type, which is consistent with the greater IAA content in the shoot tips and lower IAA content in the stems of dwarf-type pears than in standard-type pears. The CT repeat deletion in the promoter that causes a decrease in promoter activity is associated with lower *PcPIN-L* expression in the dwarf-type pears, which might limit the polar auxin transport and in turn result in the dwarf phenotype. Taken together, the results provide a novel dwarfing molecular mechanism in perennial woody plants.

## Introduction

Dwarfing and high-density planting are important developments in the cultivation and production of modern fruit orchards for higher yield per hectare and efficient mechanised management^1,2^. This is primarily due to suitable dwarf germplasm^3–5^. However, the breeding of dwarfing pear rootstocks or dwarf cultivars is still slowly developed^6^. Producing elite dwarf pear germplasm is a primary goal of pear breeders for modern pear production^7^.

‘Le Nain Vert’, which was a mutant French cultivar of *Pyrus communis*, exhibited significantly dwarf characteristics^8^. In our previous study, we obtained one dwarf seedling that showed similar features to ‘Le Nain Vert’ and designated ‘Aihuali^9^’. We crossed ‘Aihuali’ with the primary cultivar ‘Chili’ (*Pyrus bretschneideri*), and the chi-square analysis showed that the dwarf and standard character fitted a 1:1 ratio, which was controlled by a single dominant gene designated *PcDw*^9^. Subsequently, we narrowed the range of the *PcDw* gene within scaffold00074 by simple sequence repeat and single-nucleotide polymorphism markers^10^. Recently, several candidate genes were revealed by comparative transcriptome analysis^11^. However, the fundamental information about *PcDw* still remain undetermined.

Phytohormones play important roles in plant growth and development^12^. The dwarf phenotype resulted from the changes in both cell division and cell elongation, which are regulated by endogenous cytokinin (CTK), gibberellin, auxin (indole acetic acid, IAA), abscisic acid and brassinosteroid^13–16^. Amongst these crucial plant hormones, IAA is considered to be one of the most important hormones in the dwarfism mechanism. IAA is synthesised in the apical meristem and transported downward to the root tips through the cambium and phloem. This is essential for regulating the plant growth and development^17,18^. Intensive research on IAA has revealed that auxin instructs plant growth and development by regulating cell division, growth and differentiation in a concentration-dependent manner^19,20^. The differential auxin concentrations are sensed and translated into a cellular response by complex signal transduction pathways^12,21^. In apple, the common elucidation of dwarfism is the dwarfing interstems that result in less IAA transport to the root, and the abnormal distribution of IAA concentrations in turn limits the growth of the canopy^22–24^. The decreased capacity for polar auxin transport in dwarfing plants can be related to the inhomogeneous distribution of the auxin efflux carrier proteins^25–27^.

PIN-FORMED (PIN) proteins are now considered to be the auxin efflux carriers, which take part in polar cell-to-cell auxin transport; they are important basipetal transporters that transport IAA from the apical meristem to the roots^28–33^. A total of eight PIN proteins in *Arabidopsis* that can be classified into two subclasses: the plasma membrane (PM)-localised PIN1-type (PIN1, 2, 3, 4, 7) and the endoplasmic reticulum (ER)-localised PIN5-type (PIN5, 6, 8)^34–36^. The difference in the subcellular localisation appears to be functionally distinct^37,38^. A substantial amount of research revolved have been conducted to study the function of PIN1-type proteins. The *pinl-1* mutant shows a unique structure in the inflorescence axis and reduces the activity of IAA polar transport^39,40^. Mutations in the *Arabidopsis* gene *PIN3* alter differential growth of the stem and lateral root^41^. The *pin*4 and *pin*7 mutants show less inhibition of root growth than the wild type^42,43^. The auxin efflux carriers PIN3, PIN4 and PIN7 are major contributors to this auxin transport connectivity^44^. Gan *et al*. (2018) reported that the tobacco overexpressing apple *MdPIN1b* exhibits increased polar auxin transport and that lower expression of *MdPIN1b* in the M9 interstem may contribute to the dwarfing phenotype in apple trees. However, the genome and functions of PINs in pear still remain unclear^24^.

In this study, we cloned a *PcPIN-Like* (*PcPIN-L*) gene (accession number: PCP021016), as one of the candidates for *PcDw*, from the dwarf-type and standard-type pears. We found that the promoter of *PcPIN-L* in dwarf-type pears had a repeated CT deletion and resulted in lower activity of the promoter compared with that in standard-type pears. In addition, *PcPIN-L* overexpression in tobacco enhanced the growth of the stems and roots through changing the distribution of IAA. The findings of this study will clarify the role of PIN-dependent auxin polar transports in the dwarfing mechanism of pear trees and will contribute to the molecular breeding of dwarf pear cultivars, which is crucial for modern pear production.

## Results

### PCP021016: a PIN-like gene in pear

**Figure 1:**
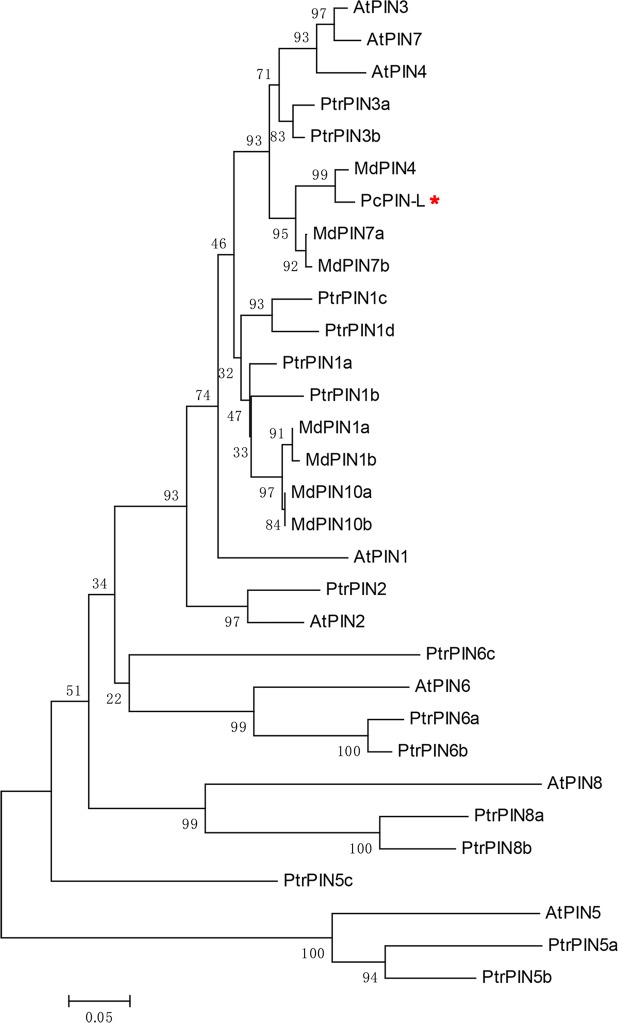
The phylogenetic tree of *PcPIN-L* with *Arabidopsis thaliana*, *Malus domestica* and *Populus trichocarpa* PINs. The phylogenetic tree was constructed using the neighbour-joining method and a bootstrap test with 1000 iterations using MEGA5.2 software on the basis of the amino acid sequences of PcPIN-L (accession no. PCP021016), AtPIN1 (AF089085), AtPIN2 (AF086907), AtPIN3 (AF087818), AtPIN4 (AF087016), AtPIN5 (AB005242), AtPIN6 (AF087819), AtPIN7 (AF087820), AtPIN8 (AL391146), MdPIN1a (EF406255), MdPIN1b (EF406256), MdPIN4 (EF406257), MdPIN7a (EF406258), MdPIN7b (EF406259), MdPIN10a (EF406260), MdPIN10b (EF406261), PtrPIN1a (Potri.012G047200), PtrPIN1b (Potri.015G038700), PtrPIN1c (Potri.006G037000), PtrPIN1d (Potri.016G035300), PtrPIN2 (Potri.018G139400), PtrPIN3a (Potri.010G112800), PtrPIN3b (Potri.008G129400), PtrPIN5a (Potri.019G052800), PtrPIN5b (Potri.013G087000), PtrPIN5c (Potri.014G146800), PtrPIN6a (Potri.005G187500), PtrPIN6b (Potri.002G072200), PtrPIN6c (Potri.001G205200), PtrPIN8a (Potri.017G078300) and PtrPIN8b (Potri.004G124200).

*PCP021016* was cloned from the dwarf-type and standard-type pears. The amino acid sequence of PCP021016 in the dwarf-type pear did not differ from the standard-type pear (Fig. S1). To further analyse the function of *PCP021016*, the amino acid sequence of PCP021016 was BLASTed in NCBI, indicating that PCP021016 had the conserved domain of the auxin efflux carrier component encoding a PIN-like gene. PCP021016 was designated *PcPIN-L*. To determine the similarity of the *PcPIN-L* sequence in relation to the PINs of *Arabidopsis thaliana*, *Malus domestica*^45^, and *Populus trichocarpa*^46^, a phylogenetic tree based on the amino acid sequences of PIN family was constructed using MEGA 5.2 software. The results showed that the PcPIN-L was most closely related to MdPIN4, MdPIN7a and MdPIN7b of *Malus domestica*, PtrPIN3a and PtrPIN3b of *Populus trichocarpa*, AtPIN3, AtPIN7 and AtPIN4 of *Arabidopsis thaliana* (Fig. 1). Multiple sequence alignment showed that the amino acid sequence of PcPIN-L was 73.0%, 66.2% and 65.9% identical with MdPIN4, MdPIN7a and MdPIN7b, 72.4%, 65.8% and 64.8% identical with AtPIN3, AtPIN7 and AtPIN4 respectively, and they all had the conserved domain of the membrane transport protein (Fig. S1). These results strongly suggested that *PcPIN-L* was a PIN-like gene in pear.

### Analysis of the *PcPIN-L* expression in dwarf-type and standard-type pears

**Figure 2:**
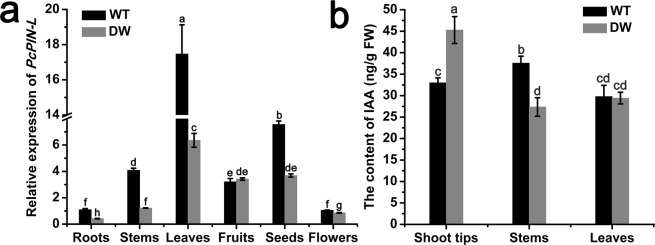
*PcPIN-L* expression and the IAA content in different tissues of dwarf-type and standard-type pears. (**a**) Relative expression of *PcPIN-L* in the roots, stems, leaves, fruits, seeds, and flowers of the dwarf-type and standard-type pears. (**b**) IAA content in the shoot tips, stems and leaves of the dwarf-type and standard-type pears. Data are the means ± SD of triplicate experiments. Different lowercase letters indicate significant differences according to Fisher’s LSD (*P* < 0.05).

The phenotype of the dwarf-type and standard-type pears was shown in Fig. S2. The dwarf-type pears showed obvious dwarf characteristics, such as compact crowns, short internodes and dwarf stature compared with standard-type pears. As one of the candidate genes for *PcDw*, the expression of *PcPIN-L* was detected in the roots, stems, leaves, fruits, seeds, and flowers of the dwarf-type and standard-type pears. As shown in Fig. 2a, the *PcPIN-L* expression level in the roots, stems, leaves, seeds, and flowers of the dwarf-type pears was significantly lower than those in the standard-type pears. Especially in the stems, the *PcPIN-L* expression level in the dwarf-type pears was only approximately 1/3 that of the standard-type pears. In the roots, leaves and seeds, the *PcPIN-L* expression level in the dwarf-type pears was less than half of the standard-type pears. In the flowers, the expression level of *PcPIN-L* in the dwarf-type pears was slightly lower than the standard-type pears (Fig. 2a). The result suggested that the lower expression of *PcPIN-L* may be an important cause of the dwarf phenotype.

We also detected the expression of *PcPIN-L* under abiotic stress and NAA treatment. It was found that the expression of *PcPIN-L* in stems and roots was significantly induced by NAA treatment and drought stress at 24 h and 48 h. However, the expression of *PcPIN-L* had no significant difference under salt and cold stress in roots, stems and leaves (Fig. S3).

### Analysis of the IAA content in dwarf-type and standard-type pears

*PcPIN-L* was an auxin efflux carrier at the PM, and the low expression of *PcPIN-L* in the dwarf-type pear would cause the heterogeneous distribution of IAA. Therefore, the IAA contents in the shoot tips, stems and leaves of the dwarf-type and standard-type pears were determined. In the shoot tips, where the IAA was synthesised, the IAA content in the dwarf-type pear was 37% higher than that in the standard-type pear. However, the IAA content in the stems of the dwarf-type pear was significantly lower than that in the standard-type pear. In addition, the IAA content in the leaves of the dwarf-type pear had no significant difference than that in the standard-type pear (Fig. 2b). This result indicated that the lower expression of *PcPIN-L* in the dwarf-type pear may have caused the heterogeneous distribution of IAA.

### *PcPIN-L* was located in the PM

**Figure 3:**
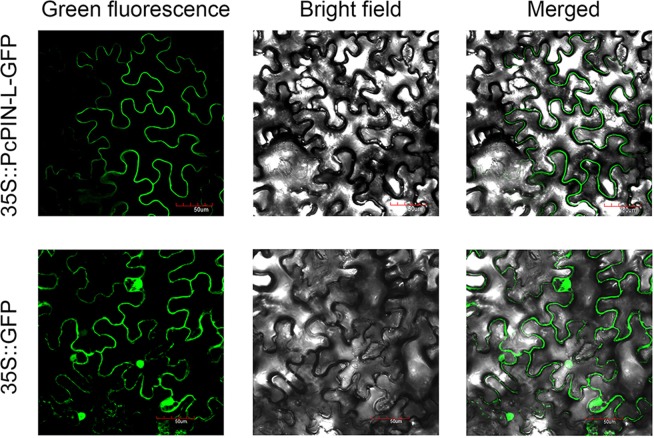
Subcellular localisation of PcPIN-L. PcPIN-L-GFP in the PM of *N. benthamiana* cells. 35 S::GFP was used as the control. The leaves were visualised by confocal microscopy (×40) three days after infiltration with *Agrobacterium tumefaciens* harbouring 35*S::PcPIN-L-GFP* or 35*S::GFP*.

*PcPIN-L* encoded a member of the PIN family and contained a conserved domain of a membrane transport protein. In *Arabidopsis*, *AtPIN3*, *4* and *7* were all PIN1-type genes, which were located in the PM^40^. To examine the subcellular localisation of PcPIN-L, GV3101 harbouring 35*S::PcPIN-L-GFP* was introduced into *Nicotiana benthamiana* leaves. Three days later, PcPIN-L-GFP fluorescence was observed exclusively in the PM, and this result contrasted with the observation of the GFP control, which showed fluorescence throughout the cells (Fig. 3).

### Cloning and analysis of the promoter region of *PcPIN-L* in dwarf-type and standard-type pears

**Figure 4:**
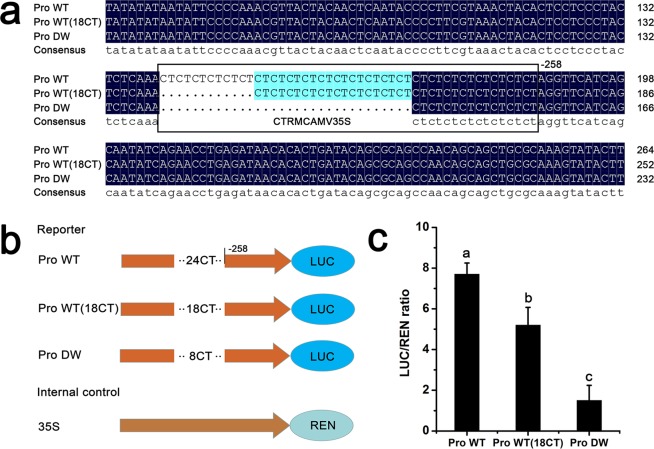
Analysis of the *PcPIN-L* promoter in the dwarf-type and standard-type pears. (**a**) Variations in the *PcPIN-L* promoter fragment between the standard-type (WT) pear and the dwarf-type (DW) pear and the promoter with 18 CT repeats. (**b**) Diagrams of the reporter and the internal control constructs used for transient expression analyses. (**c**) Observed LUC/REN ratio in transiently transformed tobacco leaves overexpressing *Pro WT*, *Pro WT* (*18CT*) and *Pro DW* fused to *pGreenII 0800-LUC* vector. Data are the means ± SD of triplicate experiments. Different lowercase letters indicate significant differences according to Fisher’s LSD (*P* < 0.05).

To determine the cause of the lower expression of *PcPIN-L* in the dwarf-type pear, we first compared the amino acid sequences of *PcPIN-L* in the dwarf-type and standard-type pears. The results showed no differences (Fig. S1). We then cloned the promoter of the dwarf-type and standard-type pears. As shown in Fig. 4a, Pro WT had 24 CT repeats at the position of −258 bp of the *PcPIN-L* promoter, whilst Pro DW only had 8 CT repeats. The dwarf-type pear had a deficiency of 16 CT repeat domains (Fig. 4a). To investigate the function of the CT repeat domain deficiency, the promoter was analysed in PlantCARE (http://bioinformatics.psb.ugent.be/webtools/plantcare/html/), and it was predicted to be a *cis*-acting element designated CTRMCAMV35S. Its function was to improve the transcription of the downstream gene.

To evaluate the transcriptional activities of the different promoters in the dwarf-type and standard-type pears, dual-luciferase reporter assay was used. The *Pro WT*, *Pro WT (18CT)* and *Pro DW* were cloned into the corresponding sites of *pGreenII 0800-LUC*. 35S::REN was used as an internal control (Fig. 4b). One-month-old *N. benthamiana* was used for the transformation. The leaves of *N. benthamiana* plants were infiltrated with GV3101. After four days transformation, the LUC and REN activities were detected. As shown in Fig. 4c, the LUC/REN ratio decreased significantly with fewer CT repeats, and the LUC/REN ratio of the Pro DW was only one fifth of the Pro WT. These combined results indicated that the lower transcript of *PcPIN-L* in the dwarf-type pear may have been caused by the CT repeat deletion in the promoter.

### Molecular marker to distinguish the dwarf-type and standard-type pear

Given the deficiency of the 32 bp CT repeat domains in the dwarf-type pear, we explored a molecular marker to distinguish the dwarf-type and standard-type pears in the F_1_ populations. Primers were designed at the two sides of the deletion domain. Five dwarf hybrids and five standard hybrids were randomly selected to verify the molecular marker. The result showed that all the stripes of the selected standard-type pear were longer than those of the dwarf-type pear, and they could correctly distinguish the dwarf-type and the standard-type pear (Fig. S4).

### Phenotype of transgenic tobacco lines overexpressing *PcPIN-L*

To investigate the function of *PcPIN-L*, we overexpressed *PcPIN-L* in tobacco. A total of seven transgenic lines were obtained (Fig. S5). The expression of *PcPIN-L* in lines 2, 3 and 7 was much higher than that in the wild type. Therefore, we selected these three lines for further analysis.

**Figure 5:**
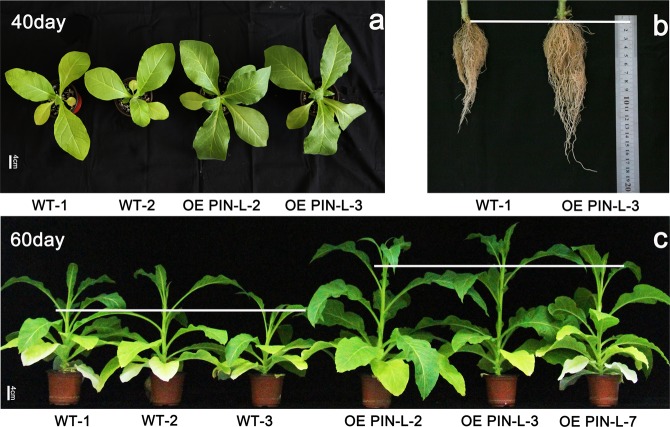
Phenotypes of the transgenic lines and control tobacco plants. Transgenic lines overexpressing *PcPIN-L* and control plants grown under greenhouse conditions after transplantation for 40 days (**a**) and 60 days (**c**). Bar = 4 cm. (**b**) Roots of the transgenic lines overexpressing *PcPIN-L* and the control plants grown under greenhouse conditions after transplantation for 80 days.

**Figure 6:**
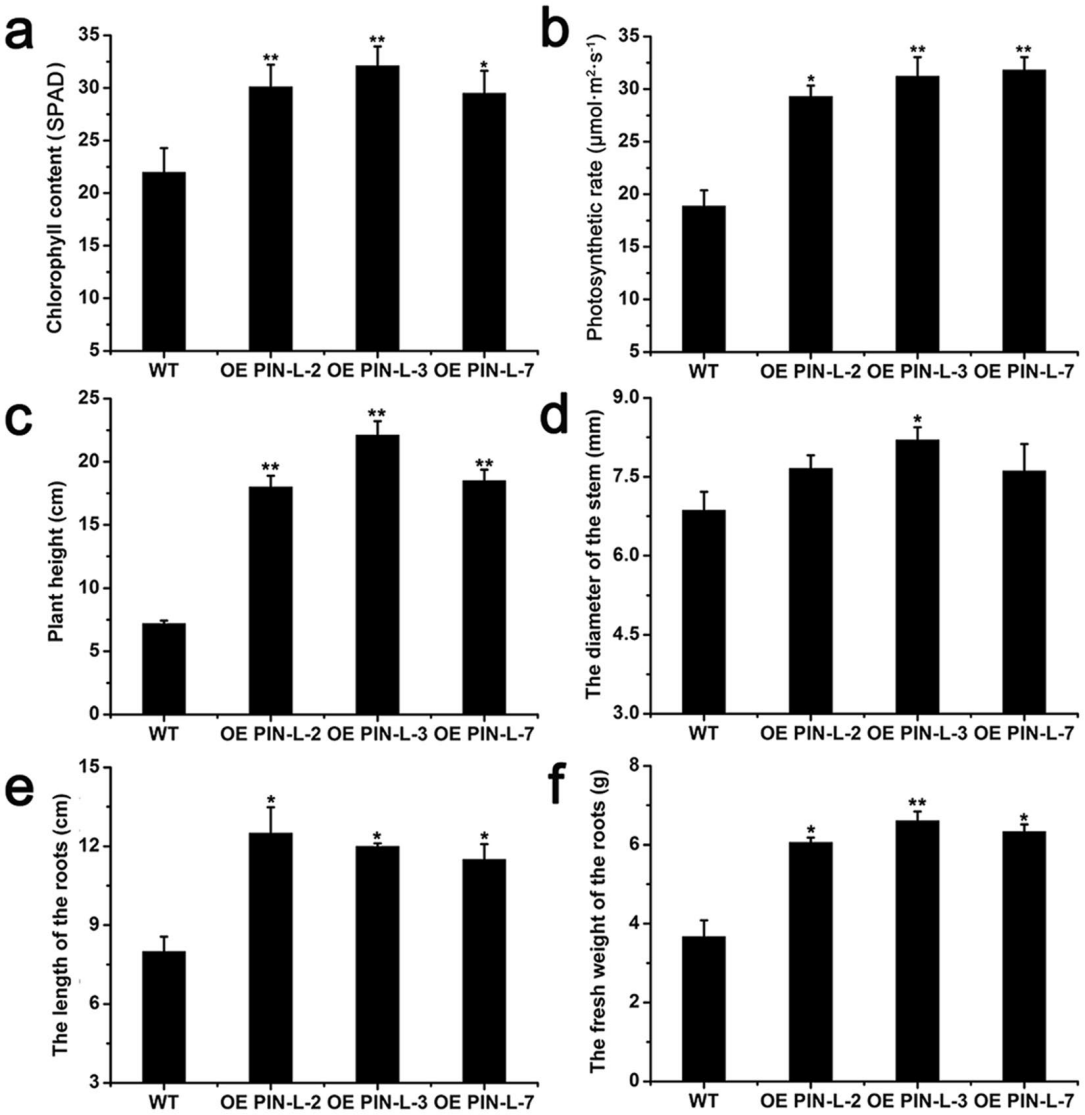
Analysis of the physiological items of the transgenic lines and control tobacco plants. (**a**) Chlorophyll content of the transgenic lines and the control plants. (**b**) Photosynthetic rate of the transgenic lines and the control plants. (**c**) Plant height of the transgenic lines and the control plants. (**d**) Diameter of the stem of the transgenic lines and the control plants. (**e**) Length of the roots of the transgenic lines and the control plants. (**f**) Fresh weight of the roots of the transgenic lines and the control plants. Data are the means ± SD of triplicate experiments. Asterisks (*) indicate significant differences from the control (Student’s *t*-test, *P < 0.05, **P < 0.01).

These three transgenic lines (2, 3 and 7) exhibited a quick growth phenotype after transplantation for 40–80 days, and the colour of the leaves of the transgenic lines was darker than that of the wild type at day 40 (Fig. 5a). After 60-day transplantation, the stem height of the transgenic lines were obviously enhanced (Fig. 5c), and the root length of the transgenic lines were much longer than wild type at day 80 (Fig. 5b). Compared with those of the wild type, the chlorophyll content and photosynthetic rate of the transgenic lines were obviously improved. The chlorophyll content of line 3 overexpressing *PcPIN-L* was 32.1 SPAD, whilst it was 21.9 SPAD in the wild type (Fig. 6a). The photosynthetic rate was also enhanced in the transgenic lines. The photosynthetic rate of line 7 was 31.8 µmol m^−2^ s^−1^, whilst that of the wild type was only 18.9 µmol m^−2^ s^−1^ (Fig. 6b). The most significant difference between the transgenic lines and the wild type was the plant height. The plant heights of the transgenic lines were all more than twice higher than that of the control plants. The plant height of line 3 reached 22.1 cm, which was three times higher than that of the wild type (7.2 cm) (Fig. 6c). The diameter of the stem was also determined, and no significant difference was observed between the transgenic lines and control plants except for line 3 (Fig. 6d). Except for the change of aerial parts, the length and the fresh weight of the roots were also significantly improved; the root length of line 2 was 12.5 cm, which was remarkably longer than that of the wild type (8 cm) (Fig. 6e). The fresh weight of line 3 was 6.61 g, which was approximately twice higher than that of the wild type (3.6 g) (Fig. 6f).

### Anatomical structure analysis of the transgenic lines overexpressing *PcPIN-L* and the wild-type tobacco

**Figure 7:**
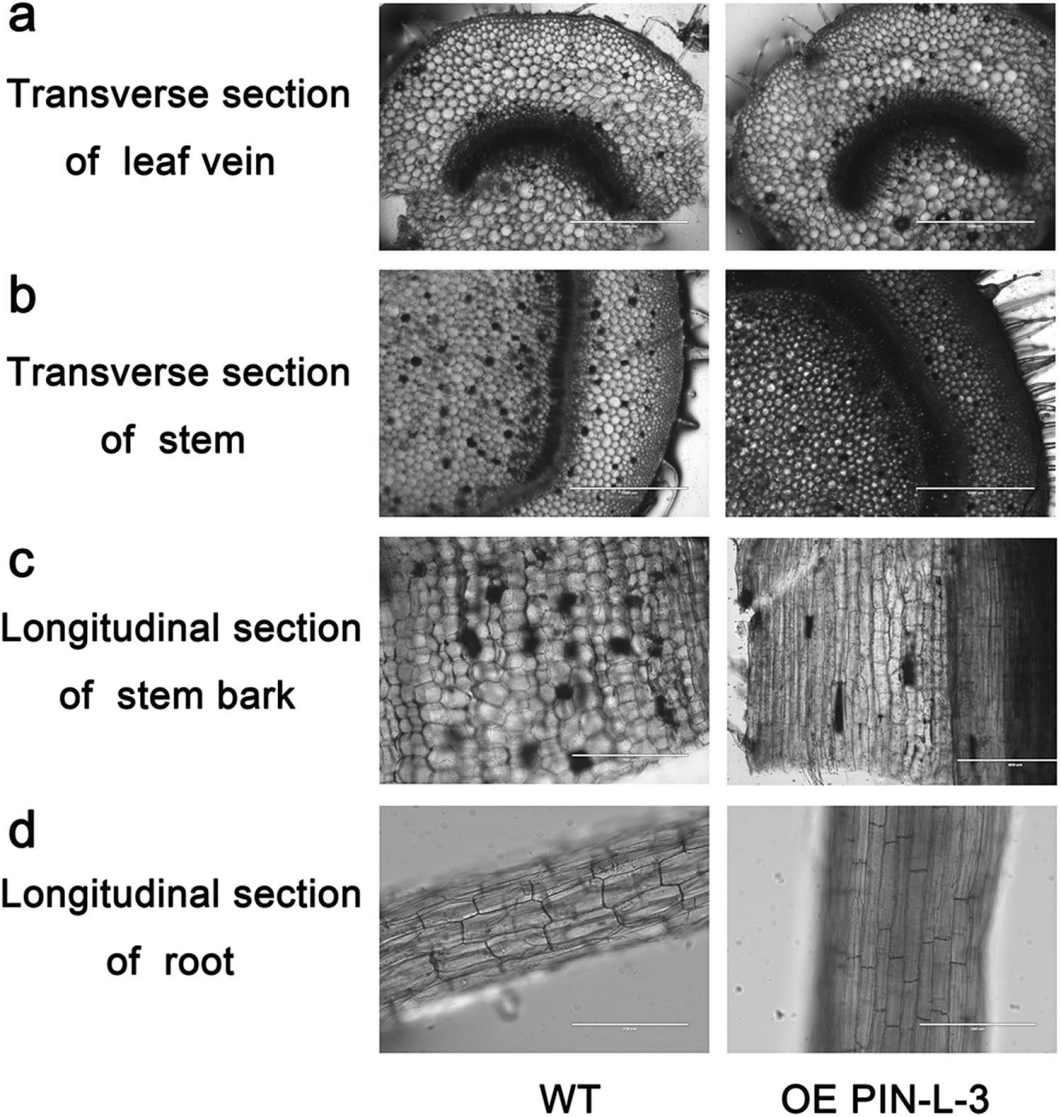
Anatomical structure analysis of the transgenic lines and control tobacco plants. (**a**) Transverse section of the leaf veins of the transgenic lines and the control plants. (**b**) Transverse section of the stems of the transgenic lines and the control plants. (**c**) Longitudinal sections of the stem bark of the transgenic lines and the control plants. (**d**) Longitudinal sections of the roots of the transgenic lines and the control plants.

The anatomical structures of the leaf veins, roots and stems between the transgenic lines and control plants were also observed. In the cross-section, no significant difference in the leaf veins was observed. However, compared with the control, the stem of transgenic line 3 overexpressing *PcPIN-L* had smaller and more cells, and the vascular bundle was more developed, indicating that the overexpression of *PcPIN-L* increased the cell division in the stems (Fig. 7a,b).

In the longitudinal section, the cells in the stem bark of the transgenic lines were longer and closer together than those in the control plants, and the elongated cells in the roots were also significantly longer in the transgenic lines than in the wild type (Fig. 7c,d). These results showed that the transgenic lines overexpressing *PcPIN-L* increased the plant height and root length primarily through enhancement of the elongation of cells in the stems and roots.

### IAA content in transgenic lines overexpressing *PcPIN-L* and the wild-type tobacco

**Figure 8:**
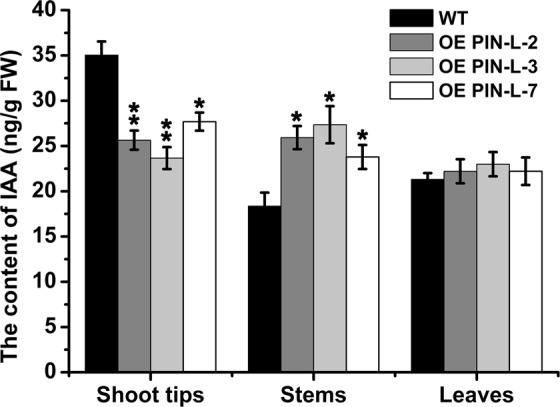
IAA content of the shoot tips, stems and leaves of the transgenic lines and control tobacco plants. Data are the means ± SD of triplicate experiments. Asterisks (*) indicate significant differences from the control (Student’s *t*-test, *P < 0.05, **P < 0.01).

Given that *PcPIN-L* is an auxin efflux carrier, its primary function is the transportation of IAA. Thus, we determined the IAA contents in the shoot tips, stems and leaves in transgenic lines and wild-type tobacco. As shown in Fig. 8, no significant difference in the IAA contents was observed in the leaves between the transgenic lines and control plants. However, the IAA content in the shoot tips was significantly lower in the transgenic lines than in the wild type, whereas the IAA content in the stem was significantly higher in the transgenic lines than in the wild type. The IAA content in the shoot tips of the wild type was 35.1 ng/g FW, whereas that in transgenic line 3 was only 23.6 ng/g FW. The IAA content in the stem of the wild type was 18.3 ng/g FW, whereas those in transgenic line 2 (25.9 ng/g FW) and 3 (27.3 ng/g FW) were approximately 1.5 times higher than that of the wild type (Fig. 8).

## Discussion

Dwarfing rootstocks and dwarf cultivars are widely used in modern fruit production for closely spaced planting and efficient mechanical management of orchards. However, dwarf cultivars and dwarfing rootstocks have some difference in dwarf mechanisms. The dwarf mechanism of the dwarfing rootstocks had been reported a lot. Firstly, the dwarfing rootstocks caused the poor connection of vascular system at the graft union, hindering the upward transportation of mineral elements and CTK^4^. Secondly, dwarfing rootstocks had weak ability to absorb water and mineral nutrients from soil and supply to scion, resulting in the dwarfing of the grafting tree^22^. Lastly, the stem of the dwarfing rootstocks obstruct the polar transportation of IAA, and this inhibited the growth of the roots and the synthesis of CTK. The decreased of the upward transportation of CTK could limit the growth of the scion, resulting in the cycle of dwarfing^24,47^. Nevertheless, the dwarf mechanism of the dwarf cultivars is still not clear, the main opinion was the change and the imbalance distribution of the plant hormones. This was caused by the difference, deletion or overexpression of hormone synthesis and transportation enzyme genes, leading to its function to be loss or abnormal, leading to plant dwarfing^48^. So far, the molecular mechanism of pear dwarfing is still unknown^15,49^.

In our previous study, we found that the dwarf growth habit, initially discovered in a French cultivar of *Pyrus communis* ‘Le Nain Vert’, exhibited monogenic inheritance determined by a dominant gene *PcDw*, and we narrowed the range of the *PcDw* gene within scaffold00074 in the pear genome^10^. A total of 49 genes were identified in this region based on the pear genome sequence. Then we combined these genes with a RNA-Seq between the dwarf-type and standard-type pears. Seven genes were significantly differentially expressed between the dwarf-type and standard-type pears, of which five (*PCP021012*, *PCP021014*, *PCP021015*, *PCP021020*, and *PCP021021*) were up-regulated and two were down-regulated (*PCP021016* and *PCP021036*)^11^. We analyzed the CDS and promoter sequence of the candidate genes between dwarf-type and standard-type pears, only to find that the CT repeat deletion in the promoter of *PCP021016* was in accordance with the dwarf phenotype. The bioinformatics analysis of PCP021016 indicated that it belongs to the auxin efflux carrier family, and IAA is considered to be one of the most important hormones in the dwarfism mechanism^13,14^. In our study, we found that the expression of *PCP021016* was significantly lower in leaves, roots, stems, seeds, and flowers of the dwarf-type pear than in the standard-type pear (Fig. 2a), and the expression of *PCP021016* was significantly induced by NAA treatment in both stems and roots (Fig. S3). Therefore, *PCP021016* was selected for further study.

The CDS domain of *PCP021016* was cloned, and the conserved domain was predicted in the CDD of the NCBI. Two conserved domains were present in PCP021016. One is 2a69, which belongs to the auxin efflux carrier family, indicating that *PCP021016* may be a PIN-like gene. Thus, *PCP021016* was designated *PcPIN-L*. The other is Mem_trans, suggesting that *PcPIN-L* could be a membrane transport protein. In *Arabidopsis*, the PIN family can be divided into two subclasses: the PM-localised PIN1-type and the ER-localised PIN5-type^35,50^. PIN1–4, 6 and 7 share a similar molecular structure in terms of the presence of a long central loop, whereas PIN5 and PIN8 possess a short central loop^33^. Different PIN proteins show different subcellular localisation^51^. The phylogenetic tree with the PIN genes from *Arabidopsis* and apple showed that *PcPIN-L* has a closer relationship with MdPIN4, MdPIN7a, MdPIN7b, AtPIN3, AtPIN7 and AtPIN4 (Fig. 1), indicating that *PcPIN-L* may have similar subcellular localisation. Subcellular localisation in tobacco leaves showed that PcPIN-L was located in the PM (Fig. 3). This was the first report on the localisation of PIN in pear. The IAA efflux carriers, which are located in the PM, are primarily responsible for the long-distance transport of IAA^24,37,38^. Thus, we hypothesised that PcPIN-L was located in the PM and might be responsible for the long-distance transport of IAA.

To detect the cause of the lower *PcPIN-L* expression in the dwarf-type pear than in the standard-type pear (Fig. 2a), we first cloned the CDS sequence of the dwarf-type and the standard-type pears and found no difference (Fig. S1). Second, the promoters of the *PcPIN-L* in the dwarf-type and the standard-type pears were cloned and sequenced. Of note, the promoter of the dwarf-type pear had a 32 bp CT deletion domain. This domain was predicted to be a CTRMCAMV35S *cis*-element by PLANTCARE, and its primary function was to improve the transcription of the downstream gene (Fig. 4a). Thus, we considered that the lower expression of *PcPIN-L* in the dwarf-type pear was caused by the deletion of the CT repeat domain. To verify this finding, we used the dual-luciferase reporter assay to detect the promoter activity with different CT repeats. We also cloned a promoter with 18 CT repeats to detect the activity compared to the dwarf-type promoter (8 CT repeats) and the standard-type promoter (24 CT repeats) (Fig. 4b). The results of transient transformation in tobacco leaves indicated that the LUC/REN activity decreased along with more deletion of the CT repeats (Fig. 4c). This suggested that the lower *PcPIN-L* expression in the dwarf-type pear might be caused by the deletion of the CT repeats. The *cis*-elements of the promoters play important roles in regulating gene expression^52,53^. In apple, Gan *et al*. (2018) reported that a *MdPIN1b* mutant allele in the promoter region upstream of the dwarfing rootstock M9 caused lower promoter activity and exhibited decreased *MdPIN1b* expression compared with the normal Baleng crab rootstock^24^. In our study, the promoters are differently mutated. We hypothesiszed that the dwarf phenotype of the pear was caused by the deletion of the CT repeats. In addition, due to the deletion of the CTRMCAMV35S *cis*-acting element in the dwarf-type pear, we also developed a molecular marker to distinguish the dwarf and standard phenotype pear in the F_1_ populations (Fig. S4).

PIN is the important outflow carrier in the polar auxin transport as it mainly mediates the polar auxin transport from the vascular tissues to root tips^29,32,54^. *PcPIN-L* is a PIN-like gene that is located in the PM, and the low *PcPIN-L* expression may affect the polar auxin transport. Therefore, we determined the IAA content in the shoot tips, stems and leaves of the dwarf-type and standard-type pears. The IAA content in the shoot tips of the dwarf-type pear was significantly higher than that in the standard-type pear, and the IAA content in stems of the dwarf-type pear was much lower than that in the standard-type pear (Fig. 2b). This result was in accordance with the IAA content in transgenic tobacco lines overexpressing *PcPIN-L*. The IAA content in the shoot tips was much lower in the transgenic lines than in the wild type, whereas the IAA content in the stem was much higher in the transgenic lines than in the wild type (Fig. 8). Given that the polar auxin transport is very important for dwarfing^15^, we hypothesised that the lower expression of *PcPIN-L* in the stem affected the IAA transportation to the lower part of the stem and the roots, which results in more IAA accumulating in the shoot tips, and the weak polar auxin transport induced the decreased growth of the dwarf pear tree.

In the trangenic tobacco lines overexpressing *PcPIN-L*. After 60 days of growth in the soil, the plant heights of the transgenic lines were all significantly higher than those of the wild type (Fig. 5). In addition, we observed the anatomical structures of the stem between the transgenic lines and control plants and found that the transgenic lines had more and longer cells in the stem (Fig. 7). This finding is consistent with the anatomical structures of the dwarf-type pear, in which longitudinal sections showed shorter vertical lengths of cortical parenchyma cells than the standard-type pear^55^. Underground, the transgenic lines developed longer and more lateral roots than the wild type (Figs. 5 and 6). We hypothesised that these changes were also related to the distribution of IAA caused by the change in polar auxin transport. Moreover, lateral root development is reported to be directly related to the polar auxin transport^42,50^. AtPIN6 is a crucial component of auxin transport and auxin homeostasis and contributes to auxin-dependent growth, such as root and shoot growth^40^. Loss-of-function *pin4* and *pin7* mutants show less inhibition of root growth than the wild type^43^.

In conclusion, we found that the deletion in the promoter of dwarf-type pear causes the lower expression of *PcPIN-L*, and *PcPIN-L* can affect the polar auxin transport and the distribution of IAA. Thus, we hypothesised that the natural CT repeat deletion in the promoter of the dwarf-type pear may have caused the downregulation of *PcPIN-L* and altered IAA basipetal transport. The IAA accumulated in the shoot tips of the pear and inhibited IAA from being transported to the lower part of the stems and roots. Finally, the low content of IAA in the stem affected its elongation resulting in the dwarf phenotype. Our study described a novel mechanism that the CT repeat deletion in the promoter of the *PcPIN-L* could cause the pear dwarf phenotype, which had never been reported before. This will contribute to develop the molecular marker for pear dwarf phenotype breeding, which can be applied in molecular breeding for producing improved dwarf cultivars.

## Materials and Methods

### Plant materials and growth conditions

‘Aihuali’ was crossed with the primary Chinese cultivar ‘Chili’ (*P. bretschneideri* Rehd.) in 2002. Five dwarf hybrids and five standard hybrids were randomly selected for the experiments. The leaves, stems, shoot tips, roots, fruits (fruit developing period), flowers and seeds were collected, immediately frozen in liquid nitrogen and stored at −80 °C for further experiment.

The tobacco seeds of the wild-type and transgenic tobacco lines ectopically expressing *PcPIN-L* were sown on soil after three-day dark treatment at 4 °C to synchronise their germination. The temperature was controlled at 24 ± 2 °C with a 12/12 h light/dark cycle, and the light intensity was approximately 200 µmol m^−2^ s^−1^.

### Cloning of the coding domain sequence (CDS) and the promoter of *PcPIN-L*

To clone the CDS of *PcPIN-L*, the total RNA of the dwarf-type and standard-type pear was isolated using an EASY Spin Plant RNA Rapid Extraction Kit (Biomed, Beijing, China). First stand cDNA was synthesized using an M-MLV Reverse Transcriptase Kit (Promega, Madison, USA). The full-length open reading frames (ORF) of *PcPIN-L* were cloned from the standard-type (WT) and the dwarf-type (DW) pear, respectively. The primers used to clone the CDS of *PcPIN-L* are shown in Table S1.

To clone the promoter of *PcPIN-L*, the DNA of the dwarf-type and standard-type pear was isolated using a Rapid Plant Genomic DNA Isolation Kit (Tiangen, Beijing, China). The promoters of *PcPIN-L* were cloned from the standard-type (*Pro WT*) and the dwarf-type (*Pro DW*) pear and ligated into the pMD19-T simple vector (Takara, Dalian, China) for sequencing. The primers used to clone the promoter of *PcPIN-L* are shown in Table S1.

### Quantitative real-time polymerase chain reaction (RT-PCR) assay

For qPCR analysis of the *PcPIN-L* expression in the dwarf-type and standard-type pears, total RNA extraction and cDNA synthesis were performed similarly using the methods described above. qRT-PCR reactions were conducted in a 20 µL volume, including 10 µL of LightCycler® 480 SYBR Green Master (Roche, Mannheim, Germany) on a LightCycler® 480 II System (Roche, Rotkreuz, Switzerland). *PcActin* (GenBank accession number: AB190176) was used as the internal control. The primer sequences for qPCR were designed on the basis of the coding sequence of *PcPIN-L*, and they are shown in Table S1.

For NAA treatment, the two-month-old pear seedlings were treated with 100 mM NAA. For abiotic stress treatments, the pear seedlings were treated with 22% polyethylene glycol 6000 (PEG 6000, for drought stress), 200 mM NaCl (for salt stress), 4 °C (for cold stress). The leaves, roots and stems of pear seedlings were collected at 0, 2, 6, 12, 24, and 48 h after treatment. To conduct qPCR analysis of the *PcPIN-L* expression in abiotic stress and NAA treatment, total RNA extraction and expression analysis were performed similarly using the methods described above.

To conduct qPCR analysis of the *PcPIN-L* expression in transgenic lines and wild-type tobacco, RNA extraction and expression analysis were performed similarly using the methods described above. *NtActin* (GenBank accession number: CAA45149) was used as the internal control. Each experiment was independently repeated three times.

### Determination of the PcPIN-L subcellular localisation

The CDS of *PcPIN-L* was subcloned into the pCAM35-GFP vector to generate a fusion protein with a green fluorescent protein (GFP) driven by the CaMV 35 S promoter. The primers are presented in Table S1. The transformation and the observation was performed as described by Zheng *et al*.^16^.

### *PcPIN-L* promoter activity of dwarf-type and standard-type pears

The promoters of *PcPIN-L* from the standard-type pear (*Pro WT*), dwarf-type pear (*Pro DW*), and the promoter with 18 CT repeats (Pro WT(18CT)) were amplified using the primers presented in Table S1, ligated into pMD19-T simple vector, digested with *Bamh*I/*Nco*I and then fused before the firefly luciferase gene in vector *pGreenII 0800-LUC*.

To detect the promoter activity of *Pro WT*, *Pro WT* (*18CT*) and *Pro DW*, the constructs were all introduced into *Agrobacterium tumefaciens* strain GV3101, respectively. The leaves of one-month-old *N. benthamiana* plants were infiltrated with *Agrobacterium* and three leaves for each assay. The promoter activities were detected four days after transient transformation using the Dual Glow assay reagents for firefly luciferase (LUC) and Renilla luciferase (REN) (Targeting Systems, San Diego, CA, USA) on a luminometer. The ratio of LUC and REN activities was used for the final quantification of the relative LUC activity. The transformation and dual-luciferase reporter assay were performed as described previously^56^. Each experiment was independently repeated three times.

### Molecular marker of the promoter of *PcPIN-L*

Given the difference of the *PcPIN-L* promoter in the dwarf-type and standard-type pears, the molecular marker was explored to differentiate the F_1_ progeny of the populations. The primers of the molecular marker are shown in Table S1. Five dwarf hybrids and five standard hybrids were randomly selected to verify the molecular marker. The DNAs of the dwarf-type and standard-type pears were isolated using the methods described above. The PCR products were detected through agarose gel electrophoresis.

### Generating transgenic tobacco plants overexpressing *PcPIN-L*

The *PcPIN-L* ORF was amplified using the primers presented in Table S1, ligated into pMD19-T simple vector, digested with *Bamh*I/*Sac*I and inserted into the pBI121 vector. The 35*S::PcPIN-L* plasmid was introduced into *Agrobacterium tumefaciens* strain GV3101. One-month-old *N. tabacum* var. NC89 was selected for transformation, which was performed as described by Wang *et al*.^57^. Transgenic plants and control plants were then verified by PCR and qPCR analysis.

### Measurements of the chlorophyll content, photosynthetic rate and observation by light microscopy in transgenic tobacco overexpressing *PcPIN-L* and the wild type

Three single homozygous insertional lines (OE PIN-L-2, OE PIN-L-3 and OE PIN-L-7) with relatively high expression of *PcPIN-L* were selected. After 80 days of growth in the soil, the plant height, stem diameter, root length and root fresh weight were measured. For the determination of photosynthetic rate, a LI-6400XT system (LI-COR, Lincoln, USA) was used. The light intensity was at 300 µmol m^−2^ s^−1^ with approximately 55% humidity at 24 °C. The chlorophyll content was detected by using the SPAD-502 chlorophyll metre. The observation of the transverse section of the leaf vein and stem and the longitudinal section of stem bark and roots was performed by light microscopy as described by Chen *et al*.^55^. Each experiment was independently repeated three times.

### Detection of the IAA content

To detect the endogenous IAA concentrations of the dwarf-type and standard-type pears, three dwarf hybrids and three standard hybrids were randomly selected for the experiments. Three replicates of 0.5 g leaf, stem and shoot tip samples were frozen in liquid nitrogen immediately after being harvested from the tree and stored at −80 °C until use. The extraction, purification and determination of endogenous levels of IAA were performed by using high-performance liquid chromatography according to the protocol described by Gan *et al*.^24^.

To detect the endogenous IAA concentrations of the transgenic lines and wild type, three transgenic lines and wild-type tobacco plants were used for the experiments. Three replicates of 0.5 g leaf, shoot tip and stem samples were used. The extraction, purification and determination of the endogenous levels of total IAA were performed similarly using the methods described above. Each experiment was independently repeated three times.

### Statistical analysis

All experiments were repeated three times according to a completely randomized design. The data were analyzed by ANOVA followed by Fisher’s LSD or Student’s t-test analysis. Statistically significant differences were indicated by P < 0.05. Statistical computations were conducted using SPSS (IBM, Armonk, NY, USA).

### Main Conclusion

The deletion in the promoter caused lower PcPIN-L expression in the dwarf-type pears, which might limit the polar auxin transport and in turn result in the dwarf phenotype.

## Supplementary information

Dataset 1
