# Supplementary material for: Deletion in the Promoter of PcPIN-L Affects the Polar Auxin Transport in Dwarf Pear (Pyrus communis L.)
Source: Sci Rep. 2019 Dec 9;9:18645. doi: 10.1038/s41598-019-55195-7 (PMC6901534; doi:10.1038/s41598-019-55195-7)
Supplement: Supplementary file 1 — Dataset 1 [file 41598_2019_55195_MOESM1_ESM.pdf]

# Deletion in the Promoter of *PcPIN-L* Affects the Polar Auxin Transport in Dwarf Pear (*Pyrus communis* L.)

**Xiaodong Zheng[1], Haiyue Zhang[1], Yuxiong Xiao, Caihong Wang, Yike Tian***

***Correspondence: Yike Tian**: tianyike6068@163.com

[1] These authors contributed equally to this work.

**1. Supplementary Figures**

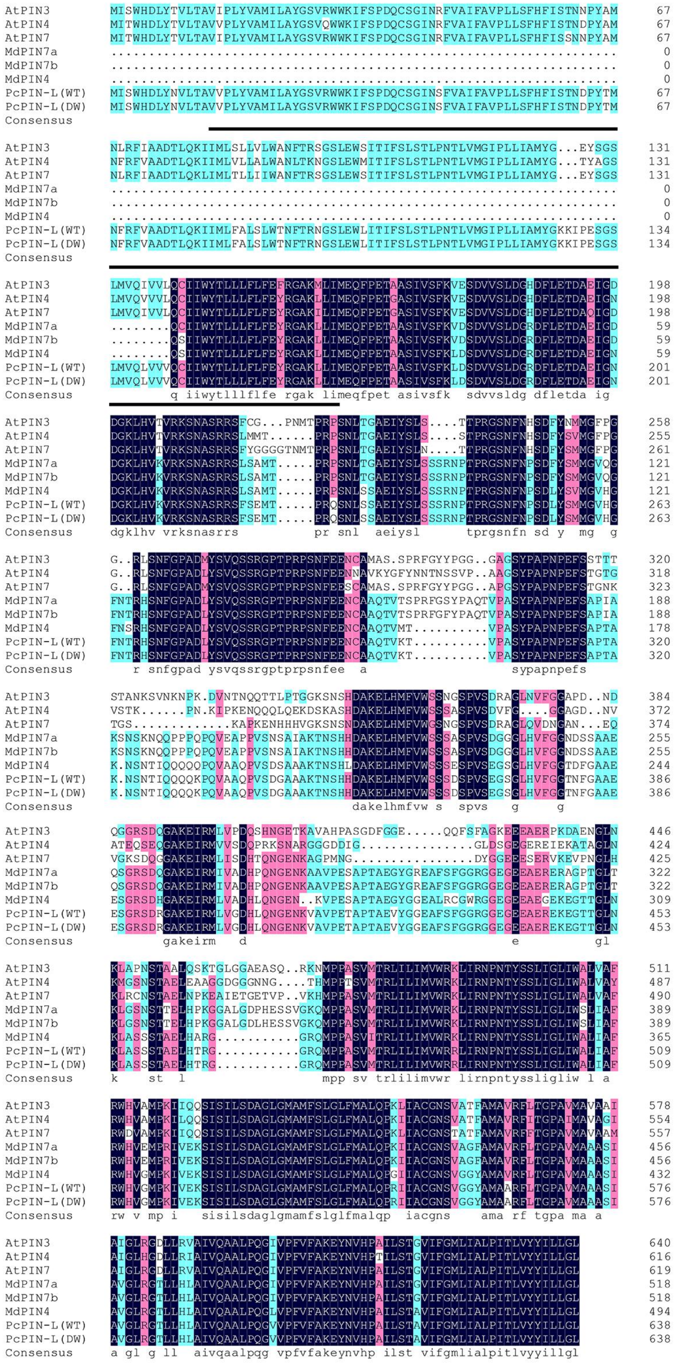

**Figure S1.** Multiple alignments were constructed using DNAMAN on the basis of the amino acid sequences of PcPIN-L (WT), PcPIN-L (DW), MdPIN4, MdPIN7a, MdPIN7b, AtPIN3, AtPIN4, and AtPIN7. The domain shared by the membrane transport protein is underlined.

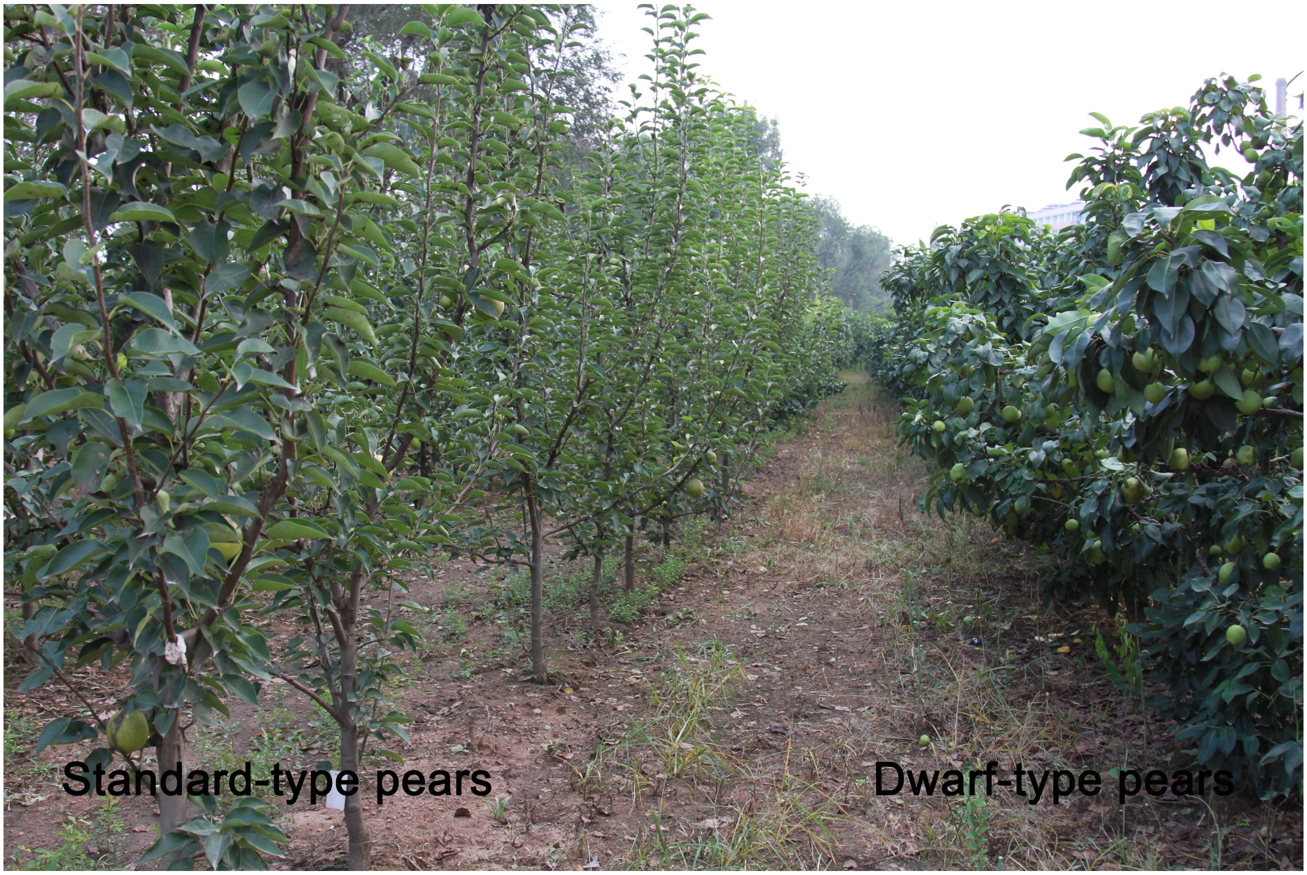


**Figure S2.** The phenotype of dwarf-type and standard-type pears. This population was planted in the field at the fruit experimental station of Qingdao Agricultural University (Laiyang, Shandong).

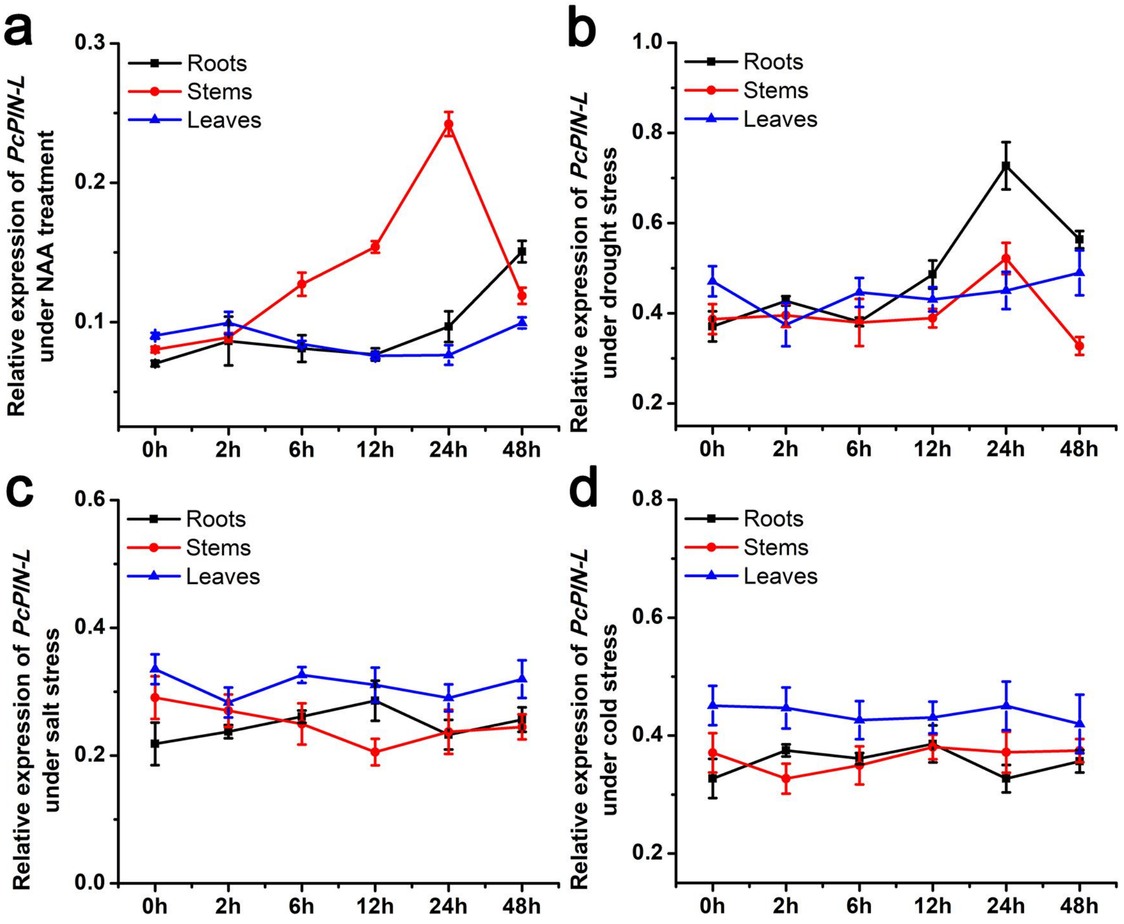


**Figure S3.** *PcPIN-L* expression under different abiotic stress and NAA treatment within 48h. Relative expression of *PcPIN-L* under NAA treatment (a), drought stress (b), salt stress (c), and cold stress (d). Data are the means ± SD of triplicate experiments.

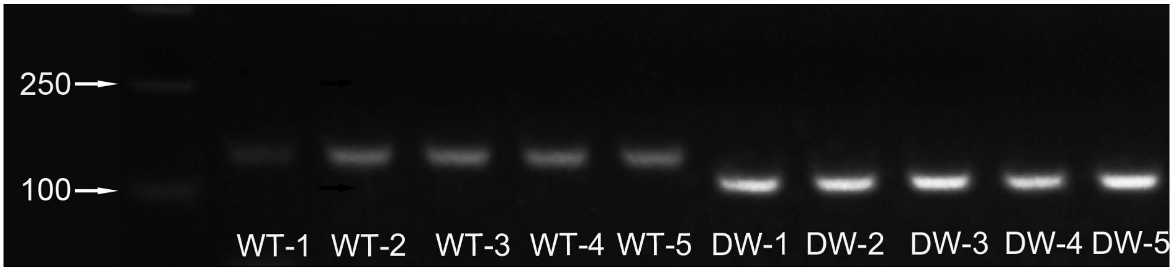


**Figure S4.** Agarose gel electrophoresis result of the DNA molecular marker used in the hybrid offspring of ‘Aihuali’ × ‘Chili’. Five dwarf-type (DW) hybrids and five standard-type (WT) hybrids were used to detect the DNA molecular marker.

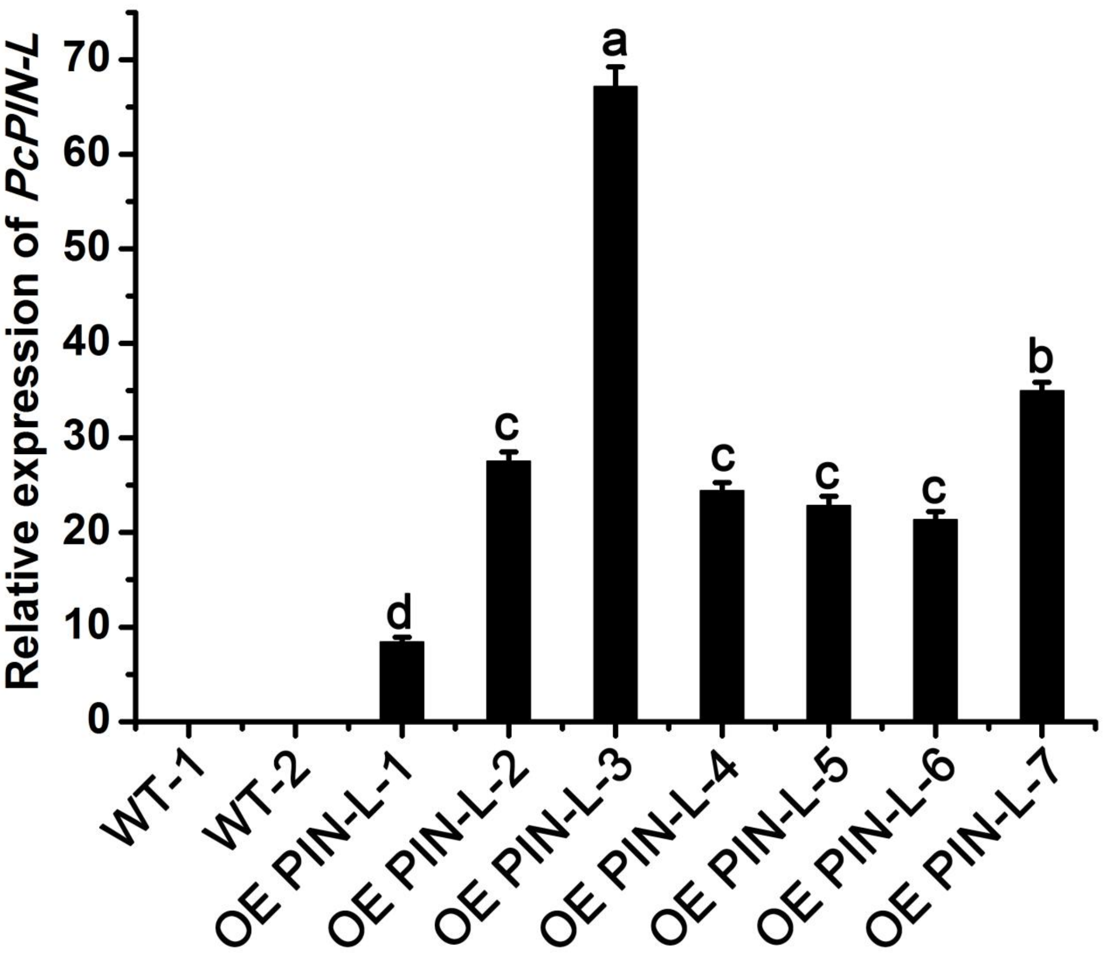


**Figure S5.** Relative expression of *PcPIN-L* in the seven transgenic lines and the control tobacco plants. Data are the means ± SD of triplicate experiments. Different lowercase letters indicate significant differences according to Fisher's LSD ($P < 0.05$).

**Table S1** The primers used for cloning, vector construction and qRT-PCR

| Primer name | Forward primer | Reverse primer | Vector |
|---|---|---|---|
| Cloning the CDS of *PcPIN-L* | ATGATTTCGTGGCACGACCT | TTACAATCCCAGAAGAATA | |
| Cloning the promoter of *PcPIN-L* | AGAAGAGAATCCTACTCCTCG | TTTTGACTGGCTGTTGGGAGT | |
| GFP-PcPIN-L | GGTACCATGATTTCGTGGCACGACCT | TCTAGACAATCCCAGAAGAATA | pCAM35-GFP |
| *PcActin* for qPCR | TGGTGTCATGGTTGGTATGG | CAGGAGCAACACGAAGTTCA | |
| *NtActin* for qPCR | AGAGGCCCTCAGACAAAC | TAGGTCCAAAGGTCACAA | |
| *PcPIN-L* for qPCR | TCCACTTTATCTCCACCAACG | AGTTGGTCCATAGGCTGAGGG | |
| Pro PcPIN-L-LUC | GGATCCAGAAGAGAATCCTACTCCTCG | CCATGGTTTTGACTGGCTGTTGGGAGT | pGreenII0800-LUC |
| PcPIN-L-pBI121 | GAGCTCCATACAGGAAGCGGCATC | GGTACCTGGGATTGGACGAAACTC | pBI121 |
| Molecular marker | CGTTACTACAACTCAATACCCC | CTGCGCTGTATCAGTGTGTT | |
